# Intake of Molecular Hydrogen in Drinking Water Increases Membrane Transporters, *p*-Glycoprotein, and Multidrug Resistance-Associated Protein 2 without Affecting Xenobiotic-Metabolizing Enzymes in Rat Liver

**DOI:** 10.3390/molecules24142627

**Published:** 2019-07-19

**Authors:** Hsien-Tsung Yao, Yu-Hsuan Yang, Mei-Ling Li

**Affiliations:** Department of Nutrition, China Medical University, 91 Hsueh-shih Road, Taichung 404, Taiwan

**Keywords:** molecular hydrogen, hydrogen-rich water, xenobiotic-metabolizing enzymes, membrane transporters, liver, oxidative stress

## Abstract

Molecular hydrogen (H_2_) has been shown to have antioxidant and anti-inflammatory activities that may reduce the development and progression of many diseases. In this study, hydrogen-rich water (HRW) was obtained by reacting hybrid magnesium–carbon hydrogen storage materials with water. Then, the effects of intake of HRW on the activities of xenobiotic-metabolizing enzymes, membrane transporters, and oxidative stress in rats were investigated. Rats were given HRW ad libitum for four weeks. The results showed that intake of HRW had no significant effect on the activities of various cytochrome P450 (CYP) enzymes (CYP1A1, 1A2, 2B, 2C, 2D, 2E1, 3A, and 4A), glutathione-*S*-transferase, and Uridine 5′-diphospho (UDP)-glucuronosyltransferase. Except for a mild lower plasma glucose concentration, intake of HRW had no effect on other plasma biochemical parameters in rats. *p*-Glycoprotein and multidrug resistance-associated protein (Mrp) 2 protein expressions in liver were elevated after intake of HRW. However, HRW had no significant effects on glutathione, glutathione peroxidase, or lipid peroxidation in liver. The results from this study suggest that consumption of HRW may not affect xenobiotic metabolism or oxidative stress in liver. However, intake of HRW may increase the efflux of xenobiotics or toxic substances from the liver into bile by enhancing *p*-glycoprotein and Mrp2 protein expressions.

## 1. Introduction

Xenobiotics, such as drugs or toxic chemicals, can be metabolized and eliminated by xenobiotic-metabolizing enzymes and membrane transporters. The liver is the major tissue responsible for detoxification of xenobiotics. The xenobiotic-metabolizing enzymes include phase I and phase II enzyme systems. The cytochrome P450 (CYP) enzymes are the major phase I enzymes responsible for the metabolism of endogenous molecules (e.g., sterols and fatty acids) and exogenous xenobiotics (e.g., drugs and toxic chemicals), resulting in the formation of more water-soluble and less toxic metabolites [1]. However, some CYP enzymes, such as CYP1A1, 3A, and 2E1, are involved in the bioactivation of chemicals, such as benzo[a]pyrene, aflatoxin B1, and acetaminophen [2,3,4], and produce electrophile intermediates that may covalently bind to proteins, lipids, and DNA. These enzyme reactions may therefore produce more reactive oxygen species (ROS) and increase oxidative damage to tissues [5]. Uridine 5’-diphospho (UDP)-glucuronosyltransferase (UGT) and glutathione S-transferase (GST) are two important phase II detoxifying enzymes that catalyze the conjugation reactions, resulting in the formation of water-soluble glucuronate and glutathione conjugates to facilitate the excretion of xenobiotics. Induction of phase II detoxifying enzymes and reduction of ROS is most pronounced in the prevention of chemical-induced tissue injuries and carcinogenesis [6]. Phase III membrane transporters include *p*-glycoprotein and multidrug resistance-associated proteins, such as Mrp2/3, which may function to shuttle xenobiotics or their metabolites across cellular membranes and to facilitate the excretion of these compounds from the liver into bile (e.g., *p*-glycoprotein and Mrp2) and blood (e.g., Mrp3) [7,8,9]. 

Molecular hydrogen (H_2_) acts as an antioxidant by selectively reducing particularly strong oxidants such as the hydroxyl radical (•OH), which can exhibit relatively stronger oxidative activities than other ROS in cells [10]. It is noteworthy that H_2_ has no cytotoxicity even at high concentrations. Consumption of hydrogen water (H_2_ dissolved in water) is a convenient, easily administered, and safe way to ingest H_2_. Hydrogen water can be made by several methods: infusing H_2_ gas into water under high pressure, electrolyzing water to producing H_2_, and reacting magnesium metal or its hydride with water [11]. Despite the low solubility of H_2_ gas in water, which can be only up to 1.6 ppm under 1 atmospheric pressure at room temperature, consumption of hydrogen-rich water (HRW) has been shown to be effective for ameliorating various diseases caused by oxidative stress in animal and clinical studies [11,12]. These findings suggest that H_2_ may be a versatile element with therapeutic activity. 

The quality of drinking water is an important health and safety information [13]. It is known that dissolved H_2_ in drinking water will gradually decrease over time. Until now, in addition to the H_2_ concentration, the quality of HRW has not been adequately described. In addition, the effects of intake of HRW on the activity of xenobiotic-metabolizing enzymes and on the membrane transporters involved in the metabolism of various drugs or chemicals are still unknown. In this study, therefore, we first assessed quality parameters of HRW, such as the dissolved H_2_ concentration, total dissolved solids (TDS), ions, and water cluster. Then, the effects of intake of HRW on xenobiotic-metabolizing enzymes, membrane transporters, and antioxidant activity in rat liver were investigated. 

## 2. Results

### 2.1. Quality of HRW

The quality of drinking water is an important information for health and safety. The quality parameters of HRW, including dissolved H_2_ concentration, oxidation-reduction potential (ORP) value, and physical and chemical properties of the water samples, are shown in Table 1. The fresh HRW contained 1550 ppb H_2_ and had a negative ORP value (control water: +293 mv; HRW: −453 mv). HRW also had a higher pH than the control water. Ca^2+^ concentrations were lower and Mg^2+^ concentrations were higher in the HRW. Other parameters of water quality, including TDS, salt, electrolytic conductivity (EC), dissolved oxygen (DO), Na^+^, K^+^, Cl^−^, and SO4^2−^ levels, showed little or no difference between the control water and HRW. 

**Figure 1:**
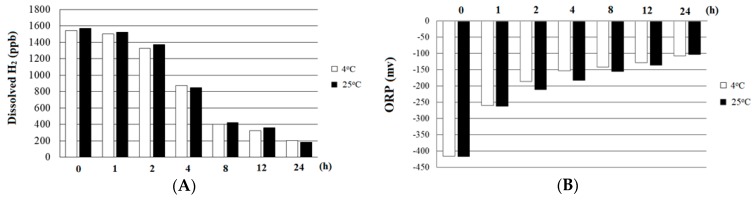
(**A**) Stability of dissolved H_2_ and (**B**) the ORP of hydrogen-rich water at 4 °C and 25 °C. ORP, oxidation-reduction potential.

The stability of dissolved H_2_ in HRW is shown in Figure 1. A H_2_ concentration of around 1500 ppb was maintained in water for 1 h, and the concentration remained around 1300 ppb for 2 h. After that, the H_2_ concentration in water gradually decreased over time. The remaining H_2_ concentration in HRW was around 200 ppb after the sample was set at 4 °C or 25 °C for 24 h. The ORP value in HRW increased gradually over time and, after 24 h, was around −100 mv. No significant differences in dissolved H_2_ or ORP values were observed in the HRW samples either at 4 °C or at 25 °C within 24 h.

**Figure 2:**
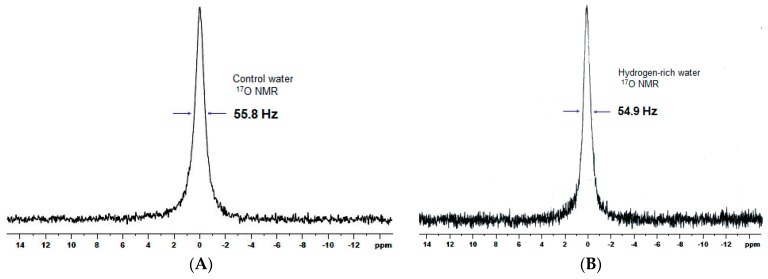
^17^O Nuclear magnetic resonance line-width of water samples: (**A**) Control water and (**B**) hydrogen-rich water.

It was noteworthy that water cluster size, determined by ^17^O NMR line-width, was comparable between the control water (55.8 Hz) and the HRW (54.9 Hz) (Figure 2). In a previous study, the water cluster size of tap water was shown to be greater than 110 Hz [14]. These results suggest that, after tap water is filtered through the first two filters, the water cluster size can be lowered by shear force. The final magnetized rod device may have strengthened the hydrogen bond network of the water and, thus, stabilized the water cluster [15].

### 2.2. Body Weight, Tissue Weight, Water Drinking Volume, and Plasma Biochemical Parameters

In this study, no significant effects were found on body weight, liver weight, kidney weight, or food intake in rats that ingested the control water or HRW for four weeks. The volume of drinking water ingested was mildly higher (+10.7%, *p* < 0.05) in the HRW group (81.8 ± 5.1 mL/day) than in the control group (73.9 ± 5.0 mL/day). The plasma biochemical parameters are shown in Table 2. Plasma glucose was mildly lower (−7.7%) in the HRW group than in the control group (*p* < 0.05). There were no significant differences in other plasma parameters including ions between the control and HRW groups.

### 2.3. Activities of Xenobiotic-Metabolizing Enzymes and Membrane Transporters

Table 3 shows the effects of intake of HRW on the activities of xenobiotic-metabolizing enzymes in the liver. No effects were found in the activity of UGT or GST in rats based on intake of HRW for four weeks. In addition, intake of HRW had no significant effect on CYP-mediated reactions.

**Figure 3:**
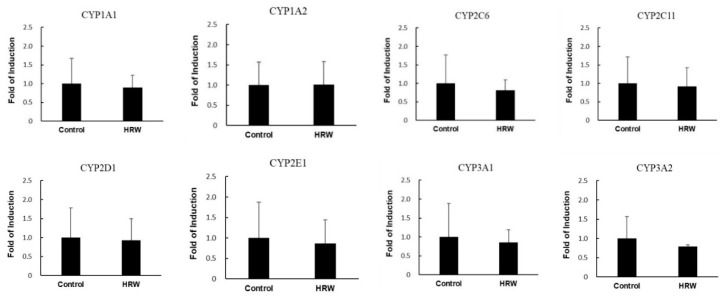
Effects of intake of HRW on the mRNA expression of various CYP enzymes in liver: The results are expressed as the mean ± S.D. of five rats.

The gene expression of CYP enzymes was also not changed (*p* > 0.05) by intake of HRW (Figure 3).

**Figure 4:**
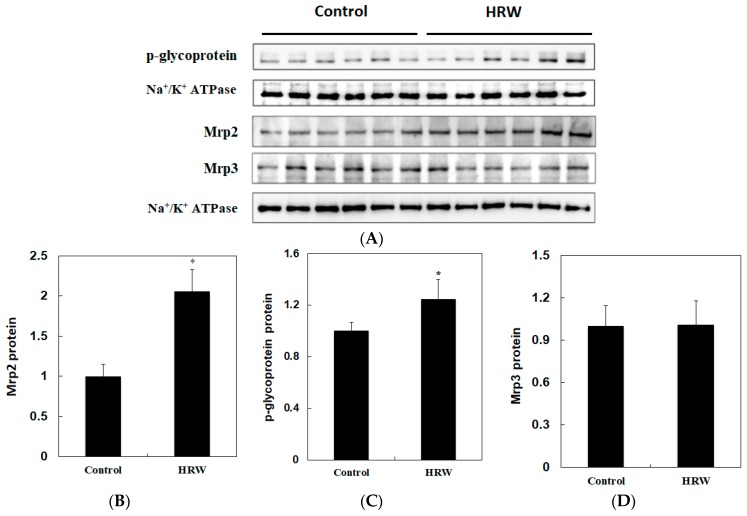
Effects of intake of HRW on *p*-glycoprotein and Mrp2/3 protein expression in liver of rats (**A**). Protein expression was determined by Western blotting. Densitometric analysis for Mrp2 (**B**), *p*-glycoprotein (**C**) and Mrp3 (**D**) protein levels corrected to each internal control is shown. The results are expressed as the mean ± S.D. of six rats. Na^+^/K^+^ ATPase acts as an internal control. The protein band was quantified by densitometry, and the level of the control was set at 1. * Significantly different from the control group at *p* < 0.05.

Immunoblots of liver membrane transporters are shown in Figure 4. Intake of HRW increased (*p* < 0.05) *p*-glycoprotein and Mrp2 protein expressions in liver. However, HRW had no significant effect on Mrp3 protein expression. These results suggest that intake of HRW may increase the efflux of endogenous substances and exogenous xenobiotics from liver into bile by increasing Mrp2 and *p*-glycoprotein protein expression without affecting the hepatic activity of xenobiotic-metabolizing enzymes in rats.

### 2.4. Oxidative Stress

As shown in Table 4, intake of HRW had no significant effects on hepatic GSH or GSSG levels, the GSH/GSSG ratio, or GSH peroxidase activity in rats compared with these animals in the control group. Also, intake of HRW did not change the hepatic TBARS value.

## 3. Discussion

Xenobiotic-metabolizing enzymes and membrane transporters are responsible for the detoxification and elimination of xenobiotics from the body. Food components can change xenobiotic metabolism by modifying these enzymes and membrane transporters. This study first showed that intake of HRW had no effect on CYP enzyme activities or antioxidant activity in rat livers. However, intake of HRW increased the efflux pumps of *p*-glycoprotein and Mrp2 in liver. These results indicate that HRW may enhance the excretion of xenobiotics from the liver into bile without altering their metabolism by xenobiotic-metabolizing enzymes. In addition, intake of HRW had no significant effect on oxidative stress in the normal physiologic condition.

CYP-mediated bioactivation and ROS formation may be responsible for chemical or drug toxicity. Increased phase II-conjugated enzymes may facilitate the elimination of xenobiotics [6]. We showed here that intake of HRW did not change the activities of CYP isozymes, UGT, and GST in the liver. Thus, intake of HRW may not change the metabolism of xenobiotics by xenobiotic-metabolizing enzymes in liver.

GSH is the most important biomolecule against ROS-induced tissue injury and can participate in the elimination of xenobiotics through GST [6]. HRW has been shown to be able to scavenge free radicals, especially hydroxyl radical (•OH), and can prevent the progression of various diseases induced by oxidative damage [11,12]. In the present study, the plasma GSH concentration and hepatic GSH, GSH/GSSG, and GSH peroxidase activities were not changed after intake of HRW. In addition, the lipid peroxidation levels in plasma and liver were not affected by HRW. Therefore, in the normal physiologic condition, intake of HRW may not affect antioxidant activity or oxidative stress in liver. These results are consistent with previous results indicating that H_2_ does not disturb normal cellular metabolic redox reactions [11].

Membrane transporters are effective pumps for elimination of conjugates of xenobiotics from hepatocytes into bile (e.g., Mrp2 and *p*-glycoprotein) [16] and plasma (e.g., Mrp3) [9]. Therefore, increased protein expressions of Mrp2/3 and *p*-glycoprotein also play roles in detoxification processes. These membrane proteins can be induced and can protect normal tissues from endogenous and exogenous toxic substances [16,17]. In the present study, Mrp2 and *p*-glycoprotein increased in the liver whereas Mrp3 did not change significantly after intake of HRW (Figure 4). Therefore, although the mechanism is still unknown, HRW may enhance the excretion of xenobiotics or endogenous toxic substances from the liver into bile and then increase their fecal excretion by increasing the efflux pumps of Mrp2 and *p*-glycoprotein. On the other hand, in this study, a little change on the concentrations of Ca^2+^, Mg^2+^, and SO4^2−^ between the control water and HRW groups did not have any influence on plasma mineral ions. A higher daily water drinking volume was found in the HRW group compared with the control group, suggesting HRW had better palatability (see descriptions in the Results section). Until now, there is a lack of evidence demonstrated that ions in drinking water may change the xenobiotic-metabolizing enzymes and transporters. Therefore, it is suggested that increased Mrp2 and *p*-glycoprotein expression in the liver after intake of HRW may be attributed to the molecular hydrogen.

Calcium and magnesium are important nutrients in the development and maintenance of human health. Supplementation with magnesium ion (Mg^2+^) from drinking water may provide substantial contributions to total intakes of Mg^2+^ in some populations and may exert beneficial effects on reducing many diseases. A protective effect of Mg^2+^ intake from drinking water has been demonstrated on reducing cerebrovascular disease and cardiovascular mortality in humans [18,19], especially in men with lower dietary magnesium intake [20]. A recent study demonstrated that Mg^2+^ added to drinking water reduces blood glucose levels by inhibiting the gluconeogenesis pathway in rat liver [21]. In this study, HRW contained a higher Mg^2+^ concentration than control water. This may be one of the explanations for the lower plasma glucose level in the HRW group, although H_2_ is regarded as an effective element to improve glucose intolerance in diabetic mice and some type 2 patients [22,23]. Therefore, HRW manufactured by reacting water with magnesium–carbon hydrogen storage hybrid materials may result in a higher Mg^2+^ concentration in drinking water and, thus, may complement daily magnesium, especially in populations with magnesium deficiency.

To date, various commercial apparatuses for HRW production (e.g., manufactured by electrolyzing water or water reacted with magnesium-containing materials) are being developed. These HRW products may have a high dissolved H_2_ concentration and H_2_ stability in drinking water. However, the quality and safety of these HRW products should be a concern (e.g., undesirable flavor and/or unknown reaction products).

In summary, intake of HRW for four weeks may not change xenobiotic-metabolizing enzymes or antioxidant activity in liver. Regular consumption of HRW may enhance detoxification process, at least in part, through an increase in the efflux of toxic substances from the liver into bile. In addition to measuring dissolved H_2_ concentration, the present study also evaluated the water quality of HRW on biological function. Because HRW is becoming more popular worldwide, the results of the present study may provide health and safety information on HRW to consumers.

## 4. Materials and Methods

### 4.1. Materials

Testosterone, ethoxyresorufin, methoxyresorufin, pentoxyresorufin, resorufin, *p*-nitrophenol, 4-nitrocatechol, NADPH, glutathione, 1-chloro-2,4-dinitrobenzene, lauric acid, 12-hydroxy lauric acid, diclofenac (sodium salt), chlorzoxazone, dextromethophen, 1,1,3,3-tetraethoxypropan, thiobarbituric acid, and heparin were obtained from Sigma (St. Louis, MO, USA). 6-β-Hydroxytestosterone was purchased from Ultrafine Chemicals (Manchester, UK). All other chemicals and reagents were of analytical grade and were obtained commercially.

### 4.2. HRW Production

**Figure 5:**
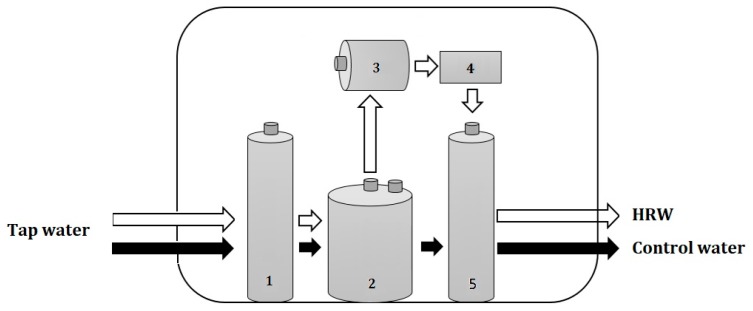
Schemes of the manufacturing process for control water and HRW from tap water: 1. Calcined ceramic filter; 2. activated carbon filter; and 3. magnesium–carbon hydrogen storage hybrid materials. Water reacted with this material and then release stable H_2_ gas; 4. activated carbon filter; 5. magnetized rod.

As shown in Figure 5, the control water (control group) was filtered from tap water (Taitung, Taiwan) by passage through a calcined ceramic filter, an activated carbon filter, and a magnetized rod (purchased from Japin biotech company, Taitung, Taiwan). The whole water devices are certified by the National Sanitation Foundation (NSF)/American National Standards Institute (ANSI) standards No. 42 (Filters are certified to reduce aesthetic impurities such as chlorine and taste/odor.), No. 53 (Filters are certified to reduce a contaminant with a health effect, which are set in this standard as regulated by the U.S. Environmental Protection Agency (EPA) and Health Canada.), and No. 401 (Treatment systems that have been verified to reduce one or more of 15 emerging contaminants, which can be pharmaceuticals or chemicals not yet regulated by the EPA or Health Canada, from drinking water (http://www.nsf.org/consumer-resources/water-quality/water-filters-testing-treatment/standards-water-treatment-systems)). The HRW was obtained from the same water apparatus except that the tap water was passed through the first two filters and then reacted with the magnesium–carbon hydrogen storage hybrid materials (Kuraray Co., Ltd., Japan) in the third device. The resulting HRW was then passed through an activated carbon filter and magnetized rod at a flow rate of 2 L/min.

### 4.3. Determinations of the Quality of HRW

The dissolved H_2_ in fresh HRW was measured with an ENH-1000 electrode (TRUSTLEX Inc, Osaka, Japan), and the oxidation-reduction potential (ORP) value was determined by use of an MP-103 electrode (Gondo Electronic Co., Ltd. Taipei, Taiwan). The stability of dissolved H_2_ and ORP values in HRW was determined at various time points, including initial (0 h), 1, 2, 4, 8, 12, and 24 h, and at 4 °C and 25 °C, respectively (Figure 1).

The other water quality parameters, including pH, total dissolved solids (TDS), electrolytic conductivity (EC), and dissolved oxygen (DO), in the experimental drinking water were determined by use of electrode equipment with a bench-top water quality meter (Chi Jui Instrument Enterprise Co., Ltd., Taiwan) (Table 1). The concentrations of cations (Na+, K+, Ca^2+^, and Mg^2+^) and anions (Cl^−^ and SO4^2−^) in the water samples were determined by inductively coupled plasma-optical emission spectroscopy (ICP-OES) (HORIBA Jobin Yvon Longjumeau, France). It is known that water quality can be influenced by ions (e.g., Na^+^, K^+^, Ca^2+^, Mg^2+^, Cl^−^, NO^3−^, and SO4^2−^ are commonly found in natural water), pH, and TDS in water and that these variables have an important influence on human health. The above water parameters can affect water clusters by the interaction of water molecules and ions or the formation of clusters of ion–water and water–water, which can change the physical properties of water (e.g., melting point) [14]. To estimate the water clustering of control water and HRW, ^17^O nuclear magnetic resonance (NMR) line-width was measured to estimate median water cluster size [14]. The wider the ^17^O NMR line-width, the larger the water cluster size. In this study, water samples were characterized by ^17^O NMR spectroscopy (Bruker 500 MHz NMR, Varian Inova, Canada). The sample (700 μL) was mixed in a 5-mm NMR spectroscopy tube and analyzed under the following conditions: 67.80 MHz, 0.202-s sampling time, 10162.6-Hz bandwidth, 4096 scans, 90° flip angle, 0.2-s relaxation delay, and room temperature (25 °C).

### 4.4. Animal Study

To investigate the effect of intake of HRW on the xenobiotic-metabolizing enzymes and membrane transporters in rat livers, sixteen male Wistar rats (aged six weeks) obtained from BioLASCO in Ilan, Taiwan were used. Rats were fed a pelleted laboratory diet with fresh control water or HRW (replaced at 5 p.m. every day) ad libitum for four weeks. The rats were all housed in plastic cages in a room kept at 23 ± 1°C with 60 ± 5% relative humidity and a 12-h light-dark cycle. At the end of the experiment, food was withdrawn for 12 h and the animals were sacrificed by exsanguination via the abdominal aorta while under carbon dioxide (70:30, CO_2_/O_2_) anesthesia. Heparin was used as the anticoagulant, and the plasma was separated from the blood by centrifugation (1750× *g*) at 4 °C for 20 min. Plasma concentrations of total cholesterol, triglyceride, alanine aminotransferase (ALT), glucose, blood urine nitrogen (BUN), creatinine, uric acid, and ions were measured immediately by use of a serum autoanalyzer (DiaSYS Diagnostic system, Germany). The liver and kidney samples from each animal were weighed and stored at −80 °C.

This study was approved (No: 2017-056) by the Institutional Animal Care and Use Committee (IACUC) of China Medical University, Taiwan. The animals were maintained in accordance with the guidelines for the care and use of laboratory animals as issued by the IACUC ethics committee.

### 4.5. Preparation of Liver Microsomes

The frozen liver was homogenized (1:4, *w*/*v*) in ice-cold 0.1 M phosphate buffer (pH 7.4) containing 1 mM ethylenediaminetetraacetic acid (EDTA). The homogenates were centrifuged at 10,000× *g* for 15 min at 4 °C. The supernatants were then centrifuged at 105,000× *g* for 60 min. The resulting microsomal pellets were suspended in a 0.25 M sucrose solution containing 1 mM EDTA and were stored at −80 °C until use. The microsomal protein concentration was determined by using a BCA protein assay kit (Pierce, Rockford, IL, USA).

### 4.6. Xenobiotic-Metabolizing Enzyme Activity Assays

The CYP enzyme activities, including methoxyresorufin *O*-demethylation (CYP1A2), ethoxyresorufin *O*-deethylation (CYP1A1), pentoxyresorufin *O*-depentylation (CYP2B), diclofenac 4-hydroxylation (CYP2C), dextromethorphan *O*-demethylation (CYP2D), *p*-nitrophenol 6-hydroxylation (CYP2E1), testosterone 6β-hydroxylation (CYP3A), and lauric acid 12-hydroxylation (CYP4A), were determined by the high performance liquid chromatography (HPLC)/mass spectrometric (MS) method [24]. Enzyme activities were expressed as pmol of metabolite formation/min/mg protein. Microsomal UGT activity was determined by using *p*-nitrophenol as the substrate, and the rate of formation of *p*-nitrophenol glucuronic acid was measured by HPLC/MS (Agilent, USA) [25]. GST was measured by the spectrophotometric method [26].

### 4.7. Determinations of GSH, GSH Peroxidase Activity, and Lipid Peroxidation

Plasma and liver homogenate were used to determine the reduced (GSH) or oxidized glutathione (GSSG) content by the HPLC/MS method as reported previously [27]. Glutathione peroxidase activity was determined spectrophotometrically according to the method of Mohandas et al. [28]. Lipid peroxidation, as measured by thiobarbituric acid reactive substances (TBARS), in plasma and tissues were assessed by the modified method of Mihara and Uchiyama [29]. 1,1,3,3-Tetramethoxypropane (Sigma, USA) was used to determine the concentrations of TBARS in the samples. Fluorescence was measured at excitation and emission wavelengths of 515 nm and 553 nm, respectively.

### 4.8. Determination of p-Glycoprotein and Mrp 2/3

Crude membrane from liver was prepared according to the method of Aleksunes et al. [30]. Each gram of liver was homogenized with 4 mL of sucrose-Tris buffer (0.25 M sucrose, 10 mM Tris-HCl, pH 7.4) containing 50 g/mL of aprotinin. The homogenate was then centrifuged at 100,000× *g* for 60 min at 4 °C. The resulting pellet was resuspended in sucrose-Tris buffer and was used for determinations of *p*-glycoprotein and Mrp2/3 by Western blot.

### 4.9. Western Blot Analysis

Equal amounts of proteins from membranes of each group were separated by SDS-PAGE and transferred to nitrocellulose membranes. The *p*-glycoprotein was purchased from Calbiochem (Darmstadt, Germany). Mrp2/3 antibodies were purchased from Abcam (Cambridge, UK). The western blot analysis was performed as described previously [24].

### 4.10. Reverse Transcription Polymerase Chain Reaction (RT-PCR) Analysis

Total RNA was extracted from homogenized liver tissue by using TRIZOL reagent (Invitrogen, Carlsbad, CA, USA) according to the manufacturer’s instructions. Total RNA (1 μg) was reverse-transcribed into first-strand cDNA by using 200 units of MMLV-RT (Promega) in a total volume of 20 μL. For real-time PCR, a SYBR system with self-designed primers and 12.5 ng cDNA was used. The self-designed primers were as follows: CYP1A1 forward: GGTTCTGGATACCCAGCTGAC; reverse: TGTGGCCCTTCTCAAATGTCC, CYP1A2 forward: GCTGTGGACTTCTTTCCGGT; reverse: TGTCCTGGATACTGTTCTTGTTGA, CYP2C6 forward: TCCTGCTGAAGTGTCCAGAA; reverse: TGCAAGGGCTGCGATGTTT, CYP2C11 forward: TGAAGGACATCGGCCAATCA; reverse: CCCATGCAACACCACAAAGG, CYP2D1 forward: ACCCATGGCTTCTTTGCTTTTC; reverse: GTCCTTGCTCCCGTACCAC, CYP3A1 forward: CTCAAGGAGATGTTCCCTGTCA; reverse: CAGGTTTGCCTTTCTCTTGCC, CYP3A2 forward: CCATCCACATCTGGTGGTCT; and reverse: TCAAAGGACGAGGACATGGTT. Amplification using 40 cycles of 2 steps (95 °C for 15 s and 60 °C for 1 min) was performed on an ABI Prism 7900HT sequence detection system (Foster City, CA, USA).

### 4.11. Statistical Analysis

Statistical differences among groups were calculated by using one-way ANOVA (SAS Institute, Cary, NC, USA). The differences were considered to be significant at *p* < 0.05 as determined by independent-sample t tests.

## 5. Conclusions

Intake of HRW for four weeks may not change xenobiotic-metabolizing enzymes and antioxidant activity in liver. Regular consumption of HRW may enhance detoxification process possibly through an increase in the efflux of toxic substances from the liver into bile.

## Figures and Tables

**Table 1:** Quality of control water and hydrogen-rich water (HRW) ^a^.

Parameters	Control	HRW
H_2_ (ppb)	0	1550
ORP (mv)	292.6	−452.7
TDS (g/L)	197.3	196.7
Salt (kU.m)	149.3	149.3
EC (ds/m)	197	197
DO (mg/L)	6.1	5.8
pH	7.6	9.3
**Ions** (ppm)		
Ca^2+^	28.0	9.1
Mg^2+^	10.2	22.8
Na^+^	4.8	5.4
K^+^	1	1
Cl^−^	2.9	3.2
SO_4_^2−^	38.5	59.8
Water cluster (Hz)	55.8	54.9

^a^ All data were measured at 25 °C. The quality of tap water was as follows: H_2,_ 0 ppb; ORP: 203.7 mv; TDS: 204.7 g/L; salt: 155 kU.m; EC: 310.7 ds/m; DO: 8.0 mg/L; pH: 8.0; Na^+^: 4.8 ppm; K^+^: 1 ppm; Ca^2+^: 51 ppm; Mg^2+^: 10 ppm; Cl^−^: 3.2 ppm; and SO_4_^2−^: 42.0 ppm. Except for water cluster, all of the above parameters were expressed as the mean value of three determinations. In the previous study, the water cluster of tap water determined by the ^17^O NMR line-width method was 113 Hz [14]. DO, dissolved oxygen; EC, electrolytic conductivity; ORP, oxidation-reduction potential; TDS, total dissolved solids.

**Table 2:** Effects of intake of HRW on plasma biochemical parameters in rats ^a^.

Parameters	Control	HRW
Total cholesterol (mg/dL)	72.0 ± 8.2	78.9 ± 9.1
Triglyceride (mg/dL)	53.9 ± 20.5	58.2 ± 13.3
Glucose (mg/dL)	155.9 ± 10.3	143.9 ± 8.1 *
BUN (mg/dL)	16.8 ± 2.4	18.8 ± 2.6
Creatinine (mg/dL)	0.31 ± 0.02	0.31 ± 0.03
Uric acid (mg/dL)	2.2 ± 0.6	2.0 ± 0.3
ALT (U/L)	44.6 ± 6.1	44.3 ± 5.1
TBARS (nmol/mL)	0.6 ± 0.1	0.6 ± 0.1
GSH (nmol/mL)	6.4 ± 2.6	6.5 ± 2.4
Na^+^ (mg/dL)	140.2 ± 1.6	144.0 ± 5.9
K^+^ (mg/dL)	6.1 ± 0.3	6.1 ± 0.3
Cl^−^ (mg/dL)	1.3 ± 0.1	1.4 ± 0.2
Ca^2+^ (mg/dL)	91.5 ± 1.1	93.3 ± 3.9
Mg^2+^ (mg/dL)	2.9 ± 0.2	3.0 ± 0.2

^a^ Results are expressed as the mean ± S.D. of eight rats in each group. ALT, alanine aminotransferase; BUN, blood urea nitrogen; GSH, reduced glutathione; TBARS, thiobarbituric acid reactive substances. * Significantly different from the control group, *p* < 0.05.

**Table 3:** Changes in hepatic drug-metabolizing enzyme activities in rats ^a^.

	Control	HRW
Phase I enzymes (pmol/min/mg protein)		
Testosterone 6β-hydroxylase (CYP3A)	682.3 ± 75.6	640.9 ± 168.1
Diclofenac 4-hydroxylase (CYP2C)	155.9 ± 10.3	147.9 ± 8.1
Dextromethorphan *O*-demethylase (CYP2D)	51.9 ± 6.1	45.2 ± 4.6
Nitrophenol 6-hydroxylase (CYP2E1)	121.1 ± 12.3	109.8 ± 15.6
Ethoxyresorufin *O*-deethylase (CYP1A1)	167.8 ± 17.2	171.5 ± 10.1
Methoxyresorufin *O*-demethylase (CYP1A2)	133.2 ± 18.2	140.6 ± 17.4
Pentoxyresorufin *O*-depentylase (CYP2B)	61.9 ± 10.4	62.3 ± 11.3
Lauric acid 12-hydroxylauric acid (CYP4A)	1524 ± 125	1488 ± 132
Phase II enzymes (nmol/min/mg protein)		
Glutathione *S*-transferase (GST)	1072 ± 175	1132 ± 88
UDP-glucurosyltransferase (UGT)	23.4 ± 2.3	25.5 ± 3.0

^a^ Results are expressed as the mean ± S.D. of eight rats in each group.

**Table 4:** Effect of intake of HRW on oxidative stress in liver ^a^.

	Control	HRW
GSH (nmol/mg protein)	4.1 ± 0.7	5.0 ± 1.4
GSSG (nmol/mg protein)	0.14 ± 0.03	0.16 ± 0.02
GSH/GSSG	29.4 ± 4.2	31.3 ± 10.2
GSH peroxidase (nmol/min/mg protein)	314.3 ± 25.6	302.0 ± 41.3
TBARS (nmol/g protein)	18.8 ± 3.6	18.9 ± 2.1

^a^ Results are expressed as the mean ± S.D. of eight rats in each group. GSH, reduced glutathione; GSSG, oxidized glutathione; TBARS, thiobarbituric acid reactive substances.

## References

[1] Rendić S. (2002). Summary of information on human CYP enzymes: Human P450 metabolism data. Drug Metab. Rev..

[2] James L.P. (2003). Acetaminophen-induced hepatotoxicity. Drug Metab. Dispos..

[3] Gonzalez F.J., Gelboin H.V. (1994). Role of Human Cytochromes P450 in the Metabolic Activation of Chemical Carcinogens and Toxins. Drug Metab. Rev..

[4] Arlt V.M., Krais A.M., Godschalk R.W., Riffo-Vasquez Y., Mrizova I., Roufosse C.A., Corbin C., Shi Q., Frei E., Stiborova M. (2015). Pulmonary Inflammation Impacts on CYP1A1-Mediated Respiratory Tract DNA Damage Induced by the Carcinogenic Air Pollutant Benzo[a]pyrene. Toxicol. Sci..

[5] Kondraganti S.R., Jiang W., Jaiswal A.K., Moorthy B. (2008). Persistent induction of hepatic AND pulmonary phase II enzymes by 3-methylcholanthrene in rats. Toxicol. Sci..

[6] Krajka-Kuźniak V. (2007). Induction of phase II enzymes as a strategy in the chemoprevention of cancer and other degenerative diseases. Postępy Hig. Med. Doświadczalnej.

[7] Kong L.L., Shen G.L., Wang Z.Y., Zhuang X.M., Xiao W.B., Yuan M., Gong Z.H., Li H. (2016). Inhibition of *p*-Glycoprotein and Multidrug Resistance-Associated Protein 2 Regulates the Hepatobiliary Excretion and Plasma Exposure of Thienorphine and Its Glucuronide Conjugate. Front. Pharmacol..

[8] Liu Y.T., Chen Y.H., Uramaru N., Lin A.H., Yang H.T., Lii C.K., Yao H.T. (2016). Soy isoflavones reduce acetaminophen-induced liver injury by inhibiting cytochrome P-450-mediated bioactivation and glutathione depletion and increasing urinary drug excretion in rats. J. Funct. Foods.

[9] Slitt A.L., Cherrington N.J., Maher J.M., Klaassen C.D. (2003). Induction of multidrug resistance protein 3 in rat liver is associated with altered vectorial excretion of acetaminophen metabolites. Drug Metab. Dispos..

[10] Setsukinai K., Urano Y., Kakinuma K., Majima H.J., Nagano T. (2003). Development of novel fluorescence probes that can reliably detect reactive oxygen species and distinguish specific species. J. Biol. Chem..

[11] Ohta S. (2014). Molecular hydrogen as a preventive and therapeutic medical gas: Initiation, development and potential of hydrogen medicine. Pharmacol. Ther..

[12] Cejka C., Kubinova S., Cejkova J. (2019). The preventive and therapeutic effects of molecular hydrogen in ocular diseases and injuries where oxidative stress is involved. Free Radic. Res..

[13] World Health Organization (2017). Guidelines for Drinking-Water Quality: Fourth Edition Incorporating the 1st Addendum.

[14] Yan Y., Ou X.X., Zhang H.P., Shao Y. (2013). Effects of nano-materials on 17O NMR line-width of water clusters. J. Mol. Struct..

[15] Szcześ A., Chibowski E., Hołysz L., Rafalski P. (2011). Effects of static magnetic field on water at kinetic condition. Chem. Eng. Process. Process. Intensif..

[16] Ayrton A., Morgan P. (2001). Role of transport proteins in drug absorption, distribution and excretion. Xenobiotica.

[17] Ghanem C.I., Gómez P.C., Arana M.C., Perassolo M., Ruiz M.L., Villanueva S.S., Ochoa E.J., Catania V.A., Bengochea L.A., Mottino A.D. (2004). Effect of acetaminophen on expression and activity of rat liver multidrug resistance-associated protein 2 and *p*-glycoprotein. Biochem. Pharmacol..

[18] Yang C.Y. (1998). Calcium and Magnesium in Drinking Water and Risk of Death from Cerebrovascular Disease. Stroke.

[19] Catling L.A., Abubakar I., Lake I.R., Swift L., Hunter P.R. (2008). A systematic review of analytical observational studies investigating the association between cardiovascular disease and drinking water hardness. J. Water Heal..

[20] Leurs L.J., Schouten L.J., Mons M.N., Goldbohm R.A., van den Brandt P.A. (2010). Relationship between tap water hardness, magnesium, and calcium concentration and mortality due to ischemic heart disease or stroke in The Netherlands. Environ. Health Perspect.

[21] Barooti A., Kamran M., Kharazmi F., Eftakhar E., Malekzadeh K., Talebi A., Soltani N. (2019). Effect of oral magnesium sulfate administration on blood glucose hemostasis via inhibition of gluconeogenesis and FOXO1 gene expression in liver and muscle in diabetic rats. Biomed. Pharmacother..

[22] Kim M.-J., Kim H.K. (2006). Anti-diabetic effects of electrolyzed reduced water in streptozotocin-induced and genetic diabetic mice. Life Sci..

[23] Kajiyama S., Hasegawa G., Asano M., Hosoda H., Fukui M., Nakamura N., Kitawaki J., Imai S., Nakano K., Ohta M. (2008). Supplementation of hydrogen-rich water improves lipid and glucose metabolism in patients with type 2 diabetes or impaired glucose tolerance. Nutr. Res..

[24] Yao H.T., Hsu Y.R., Lii C.K., Lin A.H., Chang K.H., Yang H.T. (2014). Effect of commercially available green and black tea beverages on drug-metabolizing enzymes and oxidative stress in Wistar rats. Food Chem. Toxicol..

[25] Hanioka N., Jinno H., Tanaka-Kagawa T., Nishimura T., Ando M. (2001). Determination of UDP-glucuronosyltransferase UGT1A6 activity in human and rat liver microsomes by HPLC with UV detection. J. Pharm. Biomed. Anal..

[26] Habig W.H., Jakoby W.B. (1981). Assays for differentiation of glutathione S-Transferases. Methods Enzymol..

[27] Guan X., Hoffman B., Dwivedi C., Matthees D.P. (2003). A simultaneous liquid chromatography/mass spectrometric assay of glutathione, cysteine, homocysteine and their disulfides in biological samples. J. Pharm. Biomed. Anal..

[28] Mohandas J., Marshall J.J., Duggin G.G., Horvath J.S., Tiller D.J. (1984). Low activities of glutathione-related enzymes as factors in the genesis of urinary bladder cancer. Cancer Res..

[29] Uchiyama M., Mihara M. (1978). Determination of malonaldehyde precursor in tissues by thiobarbituric acid test. Anal. Biochem..

[30] Aleksunes L.M., Scheffer G.L., Jakowski A.B., Pruimboom-Brees I.M., Manautou J.E. (2006). Coordinated expression of multidrug resistance-associated proteins (Mrps) in mouse liver during toxicant-induced injury. Toxicol. Sci..

